# Exogenous Insulin Antibody Syndrome: A Rare Cause of Extreme Insulin Resistance Treated With High-dose Corticosteroids

**DOI:** 10.1210/jcemcr/luaf175

**Published:** 2025-08-22

**Authors:** Hari Haran Rajamohan, Pierre-Nicolas Boyer, Timothy Davis, Michael Lane, Amanda Love

**Affiliations:** Department of Endocrinology, Royal Brisbane and Women's Hospital, Brisbane, QLD 4006, Australia; Royal Brisbane Clinical Unit, Faculty of Health, Medicine and Behavioural Sciences, University of Queensland, Brisbane, QLD 4006, Australia; Department of Endocrinology, Royal Brisbane and Women's Hospital, Brisbane, QLD 4006, Australia; Department of Endocrinology, Royal Brisbane and Women's Hospital, Brisbane, QLD 4006, Australia; Department of Immunology, Royal Brisbane and Women's Hospital, Brisbane, QLD 4006, Australia; Department of Endocrinology, Royal Brisbane and Women's Hospital, Brisbane, QLD 4006, Australia

**Keywords:** exogenous insulin antibody syndrome, extreme insulin resistance, type 1 diabetes mellitus, corticosteroids

## Abstract

Exogenous insulin antibody syndrome is a rare condition characterized by extreme insulin resistance caused by antibodies that neutralize the action of insulin. An 80-year-old woman with type 1 diabetes mellitus was transferred to an Australian quaternary hospital with refractory hyperglycemia and diabetic ketoacidosis despite receiving >300 units of insulin daily. Laboratory testing revealed a very high insulin antibody titer of 6758 U/mL (6758 kU/L)(<0.4 U/mL; <0.4 kU/L). Plasmapheresis was considered but was not pursued because of hemodynamic instability, multiple comorbidities and frailty. Treatment with high-dose methylprednisolone at 1 mg/kg for 5 days and 500 mg of mycophenolate mofetil twice daily, followed by a tapering regimen of prednisone, resulted in a significant improvement in extreme insulin resistance. The total daily dose of insulin after treatment returned to a baseline of approximately 70 units and insulin antibody titers were reduced by 8-fold. Remission was maintained for more than 2 years until the patient died of an aggressive squamous cell carcinoma of the buccal mucosa.

## Introduction

Exogenous insulin antibody syndrome (EIAS) is a rare cause of extreme insulin resistance (EIR) in patients exposed to exogenous insulin [1]. Berson et al described clinical hypersensitivity and insulin resistance (IR) associated with circulating insulin antibodies (IAs) in patients receiving exogenous animal insulin therapy in 1956 [2]. Before contemporary insulin preparations, immunologic IR emerged as a distinct clinical entity characterized by decreased responsiveness to large amounts of insulin. Purified and recombinant human insulin preparations have significantly reduced this phenomenon over the past few decades; yet, case reports continue to surface, with EIAS proving challenging to diagnose and treat.

## Case Presentation

An 80-year-old female from Norfolk Island was referred to an Australian quaternary hospital after a prolonged local admission for uncontrolled hyperglycemia despite escalating insulin doses. Five years before this presentation, she was diagnosed with diabetes. This diagnosis was made in the context of risk factors for both insulin resistance and deficiency, including obesity (body mass index = 31.1 kg/m^2^), exocrine pancreatic insufficiency of unclear etiology, and metabolic-associated fatty liver disease complicated by liver cirrhosis and portal hypertension. Ultimately, her diabetes was classified as type 1 diabetes mellitus based on a markedly elevated glutamic acid decarboxylase antibody titers of 747 U/mL (747 IU/mL) (<5 U/mL; <5 IU/mL) and undetectable C-peptide levels.

A year before this presentation, the patient was admitted to our center for progressively worsening ascites and significant hyperglycemia. During that admission, her total daily dose (TDD) of insulin increased to 132 units. The increase was thought to be required because of decompensated liver cirrhosis [3]. Her hyperglycemia improved with management of her liver disease. Following this and in the lead up to her recent presentation, she was stable on a basal-bolus regimen of insulin detemir (Levemir) and insulin aspart (NovoRapid) with an estimated TDD of 72 units. At the time of transfer, she had a TDD requirement of 372 units as a subcutaneous basal-bolus regimen, along with intravenous insulin infusion, without achieving inpatient glycemic targets.

## Diagnostic Assessment

The patient arrived via air transfer in a stable condition. She was afebrile, and her vital signs were within normal limits. She was alert and oriented, without signs of hepatic encephalopathy. She weighed 65 kg and was 1.65 m tall (body mass index = 23.8 kg/m^2^). Notably, she had lost 20 kg since her previous admission. There was evidence of proximal myopathy with thinning of skin and easy bruising, lipohypertrophy in the abdomen, and moderate ascites.

Laboratory testing revealed a fasting blood glucose level of 385.2 mg/dL (21.4 mmol/L)(70-100 mg/dL; 3.9-5.6 mmol/L), with undetectable C-peptide levels. Her hemoglobin A1C was 8.3% (67 mmol/mol)(4.3%-6.0%; 23-42 mmol/mol), suggesting an acute or subacute deterioration over weeks rather than chronic deterioration, appreciating that her liver disease may have resulted in a falsely lower hemoglobin A1c level [4]. Liver enzyme derangement with hyperbilirubinemia was evident on biochemistry (Table 1) and was stable compared to the recent baseline. Computed tomography of the abdomen and pelvis revealed features of cirrhosis and portal hypertension, including moderate ascites.

**Table 1.** Relevant laboratory results

Test	Results	Reference range
**Full blood count**		
Hemoglobin	12.5 g/dL (125 g/L)	11.0 g/dL-16.5 g/dL (110-165 g/L)
White blood cell count	4500/μL (4.5×10^9^/L)	3500-11000/μL (3.5-11.0×10^9^/L)
Platelets count	175 × 10^3^/μL (175 × 10^9^/L)	140-400 × 10^3^/μL (140-400 × 10^9^/L)
**Liver function panel**		
Prothrombin time	12 seconds	9-13 seconds
INR	1.0	0.9-1.2
Albumin	**2.60 g/dL** **(26 g/L)**	3.50-5.50 g/dL (35-50 g/L)
Bilirubin (total)	**1.87 mg/dL** **(32 µmol/L)**	<1.00 mg/dL (<20 µmol/L)
Bilirubin (conjugated)	**0.76 mg/dL** **(13 µmol/L)**	<0.30 mg/dL (<5.1 µmol/L)
ALP	**319 U/L** **(5.33 μkat/L)**	30-110 U/L (0.50-1.84 μkat/L)
GGT	**343 U/L** **(5.73 μkat/L)**	<38 U/L (<0.63 μkat/L)
ALT	21 U/L (0.35 μkat/L)	<34 U/L (0.57 μkat/L)
AST	**33 U/L** **(0.55 μkat/L)**	<31 U/L (0.52 μkat/L)
**Endocrine panel**		
TSH	1.73 mIU/L (1.73 IU/L)	0.55-4.78 mIU/L (0.55-4.78 IU/L)
Cortisol (morning)	18.15 μg/dL (507 nmol/L)	5.07-23.20 μg/dL (140-640 nmol/L)
HbA1C	**8.3%** **(67 mmol/mol)**	4.3-6.0% (23-42 mmol/mol)
C-peptide	**<0.3 ng/mL** **(<0.10 nmol/L)**	1 hour post glucose load: 5.0-12.0 ng/mL (1.65 nmol/L-3.96 nmol/L)
Insulin antibodies	**6758 U/mL** **(6758 kU/L)**	<0.4 U/mL (<0.4 kU/L).

Abnormal values are shown in bold font. Values in parentheses are International Systems of Units (SI).

Abbreviations: ALP, alkaline phosphatase; ALT, alanine aminotransferase; AST, aspartate aminotransferase; C-peptide, connecting peptide; GGT, gamma-glutamyl transferase; HbA1c, hemoglobin A1c; INR, international normalized ratio.

She remained hyperglycemic despite being on a continuous intravenous insulin infusion running at 10 units/hour and later developed diabetic ketoacidosis on 18 units/hour of insulin. Over the next few days, her insulin requirements escalated to 800 units/day without achieving euglycemia.

In the context of such EIR, EIAS was suspected. Testing of IAs was conducted via ELISA with a result of >50 U/mL (50 kU/L)(<0.4 U/mL; <0.4 kU/L). The result was confirmed with a second sample. The laboratory also performed further quantitative studies using an insulin-binding capacity assay to assess the response to treatment (Table 2).

**Table 2.** Changes in insulin antibody levels and insulin requirements following high-dose corticosteroids

Date	Final total insulin antibody levels	Insulin antibody post competition with cold Actrapid	Reference range	Insulin requirements (units)
Day 1	6758 U/mL (6758 kU/L)	65.3 U/mL (65.3 kU/L)	<0.4 U/mL (<0.4 kU/L)	251
Day 7	8781 U/mL (8781 kU/L)	61.9 U/mL (61.9 kU/L)	<0.4 U/mL (<0.4 kU/L)	677
**MMF 500 mg BD started on day 14 and methylprednisolone commenced on day 16**				
Day 41	9088 U/mL (9088 kU/L)	43.6 U/mL (43.6 U/mL)	<0.4 U/mL (<0.4 kU/L)	132
**The patient's prednisone dose was weaned over 3 months, and she was only on 5 mg on day 123.**				
Day 123	842 U/mL (842 kU/L)	3.7 U/mL (3.7 kU/L)	<0.4 U/mL (<0.4 kU/L)	70

Values in parentheses are International Systems of Units (SI).

Abbreviations: BD, twice per day; MMF, mycophenolate.

The IA assay results took several weeks to process, during which time other causes of EIR were also considered. Infection was considered, given the presence of subjective fevers in the preceding weeks, along with a mildly elevated C-reactive protein level of 46 mg/L (4.6 mg/dL)(<5.0 mg/L; <0.5 mg/dL). However, an extensive workup yielded unremarkable results. Endogenous Cushing syndrome was considered. A 1-mg overnight dexamethasone suppression test done under suboptimal inpatient conditions failed to suppress, with a day 2 cortisol level of 188 nmol/L (6.81 μg/dL)(<50 nmol/L; <1.81 μg/dL). Further testing for the possibility of cortisol excess was postponed until the patient stabilized, but then a decision was made to start high-dose prednisone for EIAS.

Insulin malabsorption resulting from lipohypertrophy was excluded with the use of intravenous insulin. Cirrhosis was also considered as hepatic damage and portal-systemic shunting impair glucose absorption and glycogen storage, resulting in hyperglycemia [3]. However, the patient exhibited more pronounced IR than expected for liver cirrhosis.

## Treatment

**Figure 1.**
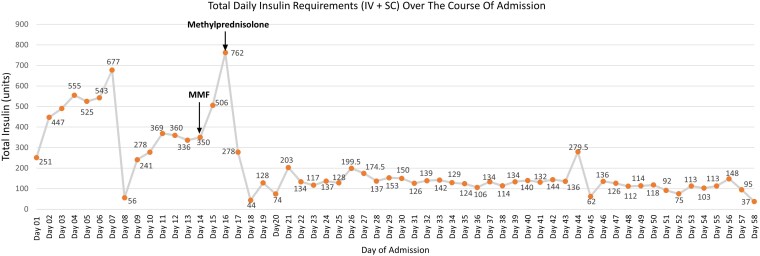
The patient received oral mycophenolate mofetil on day 14 of admission, followed by intravenous methylprednisolone on day 16 of admission, with a resultant decline in total daily insulin requirement (including intravenous and subcutaneous insulin) from 762 units/day to 44 units/day, with an average total daily insulin requirement of approximately 119.5 units/day over the following weeks.

The patient was managed collaboratively by internal medicine, endocrinology, and immunology. She began treatment with mycophenolate mofetil (MMF) 500 mg twice daily and high-dose intravenous methylprednisolone with rapid improvement in hyperglycemia and insulin requirement over 48 hours (Fig. 1). The TDD shown for each day of admission in Fig. 1 was the amount needed to maintain a blood glucose level between 8 and 12 mmol/L (144-216 mg/dL), a target set to acknowledge the risk of potential hypoglycemia in the context of the IAs, her liver disease, and frailty. By day 3 posttreatment, the patient's daily insulin requirement had fallen from 762 units to 44 units and remained stable a month after therapy initiation. This confirmed that EIAS was the main process responsible for the EIR. She was transitioned to regular oral prednisone 50 mg, which was tapered by 10 mg every 2 weeks. MMF was gradually increased to 1 g twice daily. The patient was changed to insulin lispro (Humalog), which has a lower immunogenic potential than Insulin aspart (Novorapid) [5]. As hyperglycemia improved, the patient experienced recurrent nocturnal and early morning hypoglycemia. This was managed by adjusting her insulin regimen and dividing her prednisone dose into morning and evening doses. The patient also began continuous glucose monitoring and received education on preventing and treating hypoglycemia.

## Outcome and Follow-up

The patient was discharged after a 2-month hospital stay. A month following her discharge, she presented with COVID-19. During this admission, the patient's MMF was temporarily withheld. At the time of the presentation, she was taking 15 mg of prednisone daily. This infection caused a recurrence of significant hyperglycemia and an increase in her insulin requirements. Consequently, her prednisone dose was temporarily increased to 50 mg for 5 days, resulting in an improvement in her hyperglycemia, suggesting a flare of EIAS in the context of infection. After this admission, she continued weaning her prednisone. Over the next 16 months, the patient was monitored remotely using continuous glucose monitoring and telehealth to Norfolk Island. She remained in remission from EIAS. Her daily insulin and prednisone doses were reduced to 70 units and 5 mg, respectively. She continued to take MMF at 1 g twice daily. Two years after her index EIAS admission, she developed a buccal squamous cell carcinoma. She underwent palliative radiotherapy. MMF was discontinued and she commenced rituximab, which maintained remission. Unfortunately, the buccal squamous cell carcinoma continued to progress, causing severe pain and reduced oral intake. She was admitted to a palliative care unit for supportive care and died 27 months after the diagnosis of EIAS was made.

## Discussion

EIR is defined as requiring more than 3 units/kg of insulin [6]. The causes of EIR include medications, endocrine disorders, lipodystrophies, physiological factors, pseudo-resistance, and IR syndromes. It is rarely an immune-mediated phenomenon where exogenous insulin induces IAs. The prevalence of EIAS and IA titers is higher in patients with type 1 diabetes mellitus compared to those with type 2 diabetes mellitus [7].

Despite performing the IA assay at the only Australian laboratory offering high-quality and qualitative testing, we acknowledge its limitations for guiding management. The 20-day turnaround time for the results hindered real-time decision-making and assessment of response to therapy. Furthermore, IA titers remained elevated even after commencing treatment, despite improvements in the patient's clinical status. Overall, as IA measurements exhibit considerable variability and correlate poorly with clinical status [8], we relied on glycemic control and insulin requirements to evaluate the effectiveness of treatment.

Our literature review identified 15 case reports published between 1990 and 2021, which are summarized in Table 3. The following criteria were applied to our literature search:

**Table 3.** Summary of literature review

Patient information (age/sex)	Px	Type of insulin	Pre-Tx insulin requirement	Tx	Post-Tx insulin requirement	Country	Reference
53 M	DKA + Urticaria	HRI	>200 U/day	CS	65 U/day	USA	Ganz (1990) *Journal of Allergy and Clinical Immunology* [9]
58 M	DKA B: LADA	NS	85 U/day	CS	35 U/day	Taiwan	Chang (1990), *Chinese Medical Journal* [10]
55 M	DKA B: T1DM	HRI	5.63 U/kg/day	CS + AZA + PLEX	0.66 U/kg/day	Yugoslavia	Micić (1993) *Diabetes Research Clinical Practice* [11]
27 M	DKA B: T1DM	NPH and INH	>300 U/day	Insulin changed to Insulin lispro	58 U/day	Finland	Lahtela (1997) *Diabetes Care* [12]
45 M	High BGL B: T2DM, Liver dysfunction	NPH and INH	120 U/day	Insulin ceased +OHG	Nil insulin required	Japan	Hara (2000) *Journal of Internal Medicine* [13]
54 F	High BGL B: T1DM	Human Insulin	60 U/day	Insulin changed to Insulin lispro f/b Insulin cessation	Nil insulin required	Japan	Asai (2003) *Diabetes Research Clinical Practice* [5]
12 M	High BGL B: T1DM	Human Insulin	6000 U/day	Insulin changed to insulin lispro + CS + MMF	250 U/day	UK	Segal (2008) *Pediatric Diabetes* [14]
68 F	High BGL B: T2DM, Obesity	Novomix 30	>300 U/day	Insulin changed to insulin lispro and insulin glargine + PLEX + MMF + IVIG	Died	UK	Greenfield (2009) *Diabetic Medicine* [15]
48 M	DKA + Hypo B: T1DM	Insulin aspart and insulin glargine	NS	Insulin changed to insulin glulisine	20 U/day	Japan	Yanai (2011) *Diabetes Care* [16]
62 M	DKA B: T1DM, ccRCC	Insulin lispro and insulin detemir	72 U/day	Insulin changed to insulin glulisine and insulin glargine + CS + DFPP	32 U/day	Japan	Wade (2017) *Renal Replacement Therapy* [17]
65 M	High BGL B: T2DM	Humulin U-500®	930 U/day	CS f/b rituximab + MMF	325 U/day	USA	Hao (2017) *Diabetes Care* [18]
43 M	High BGL + DKA B: T2DM	Insulin aspart and insulin glargine	300 U/day	Insulin changed to insulin glulisine, insulin aspart and insulin glargine + CS + PLEX + IVIG f/b discharge with CS + AZA + HCQ	75-80 U/day	China	Zhuang (2020) *Journal of Clinical Apheresis* [19]
70 M	DKA B: T2DM, Psoriasis	Regular insulin, INH and NPH	>9 U/kg/day	CS + PLEX + MMF	170 U/day	India	Ahmed (2021) *Diabetes Technology and Therapeutics* [20]
51 F	DKA B: T1DM	Insulin aspart and insulin detemir	>400 U/day	Insulin changed to Humulin U500 (CSII) + MMF	40 U/day	USA	Jerkins (2021) *Diabetes Therapeutics* [21]
61 M	Recurrent DKA B: T2DM	Insulin degludec and Humulin 500	>8000 U/day	MMF + PLEX	195 U/day	USA	Brooks (2021) *AACE Clinical Case Reports* [22]

Abbreviations: AZA, azathioprine; B, medical background; BGL, blood glucose levels; ccRCC, clear cell renal cell carcinoma; CS, corticosteroids; CSII, continuous subcutaneous insulin infusion; DFPP, double filtration plasmapheresis; DKA, diabetic ketoacidosis; F, female; f/b, followed by; HRI, human recombinant insulin; Hypo, hypoglycemia; HCQ, hydroxychloroquine; INH, insulin neutral human; IVIG, intravenous immunoglobulin; LADA, latent autoimmune diabetes of adult; M, male; MMF, mycophenolate mofetil; NPH, neutral protamine hagedorn insulin; NS, not specified; PLEX, plasma exchange; Px, presentation; T1DM, type 1 diabetes mellitus; T2DM, type 2 diabetes mellitus; Tx, treatment; +, in addition.

Inclusion criteria:

- Predominant clinical syndrome of EIR
- Patients treated with recombinant human insulin or insulin analogue

Exclusion criteria:

- Patients only treated with nonhuman insulin
- Anti-insulin receptor antibody (type B IR syndrome)
- The predominant clinical syndrome is hypoglycemia

Finding clear guidance on management from case reports was challenging because of the heterogeneity of conditions, ages, and comorbidities. Clinicians in some of these case reports had limited access to reliable IA assays, so it is unclear whether IR was predominantly due to IAs. As the condition often self-resolves within 3 to 6 months, milder cases of EIAS may have been overlooked. This could have led to publication bias, where only novel strategies with increasingly challenging cases were reported.

Based on the limited evidence, we initially planned to treat with MMF and plasmapheresis. Due to concerns of an event related to patient frailty, plasmapheresis was deferred and ultimately avoided after response to other therapies. Rituximab was also considered; however, contemporaneous literature advised caution regarding B-cell-depleting therapy during the COVID-19 pandemic [23]. The urgency of the situation led to the decision to initiate high-dose steroids following a risk-benefit discussion with the patient. Following a short course of intravenous methylprednisolone, corticosteroid dosing followed a published regimen for an alternative antibody-mediated disease [24].

The use of glucocorticoids has been limited in literature because they can be counterproductive, exacerbating IR. The exact mechanism of action of glucocorticoids on EIAS is unclear. It is hypothesized that they inhibit IA production and/or promote the dissociation of IA immune complexes, leading to an improvement in IR [1]. The patient's recurrent early morning and nocturnal hypoglycemia following commencement of therapy was attributed to this dissociation of IA complexes, which primarily occurs at night and during fasting episodes [25].

Literature lacks guidance on the timing of immunosuppression cessation. Due to the morbidity stemming from 2 prolonged hospital admissions for EIAS, there was reluctance to stop MMF and low-dose prednisone. These concerns were confirmed during the patient's second admission with COVID-19, when withholding MMF resulted in a significant increase in insulin requirements of up to 200 units. The low-dose prednisone was also continued because of suspected adrenal insufficiency from long-term steroid use, along with the patient's preference to remain on this regimen instead of switching to multiple daily doses of short-acting glucocorticoids.

The case highlights the challenges of balancing the risk of EIAS recurrence with the adverse side effects of long-term immunosuppression. Guidelines around the duration and selection of appropriate immunosuppressants are required as more cases of EIAS emerge.

## Learning Points

- Clinicians should have a high index of suspicion for EIAS in EIR in patients exposed to exogenous insulin
- Treatment consists of immunosuppression, which counterintuitively often includes high-dose corticosteroids in the acute setting.
- Clinicians should exercise caution with treatment selection, especially when the patient is elderly, has comorbidities and is frail
- Clinicians should be cognizant of the negative impact of long-term immunosuppression in EIAS.

## Data Availability

Data sharing is not applicable to this article as no datasets were generated or analyzed during the current study.
